# Controlled bile acid exposure to oesophageal mucosa causes up-regulation of nuclear γ-H2AX possibly via iNOS induction

**DOI:** 10.1042/BSR20160124

**Published:** 2016-07-08

**Authors:** Bo Jiang, Shengqian Zhao, Zhen Tao, Jin Wen, Yancheng Yang, Yin Zheng, Hongling Yan, Ying Sheng, Aimin Gao

**Affiliations:** * Department of Pathology, The 159th Hospital of PLA, Zhumadian, Henan 463000, China; † Department of Neurology, The General Hospital of Jinan Military Command of PLA, Jinan, Shandong 250031, China

**Keywords:** dysplasia, oesophageal adenocarcinoma, genotoxic stress, metaplasia, metaplasia, preneoplastic lesion

## Abstract

Using an *in vitro* model in which flatmounts of oesophagus was periodically exposed to bile acids, we demonstrate, using multiple methods, that the bile acid receptor TGR5, inducible nitric oxide synthase (iNOS) and γ-histone family 2A variant (γ-H2AX) are up-regulated. This indicates that bile acids cause up-regulation of iNOS, which further causes genotoxic stress as evidenced by increase of the highly sensitive marker, phosphorylated histone. *In vitro* nitric oxide (NO) assays showed increased production of nitric acid in the oesophageal epithelium exposed to the bile acids. This increase was inhibited in the presence of the nonspecific iNOS inhibitor aminoguanidine (AG). Cumulatively, the results of the present study provide suggestion that not only acid reflux, but also non-acid reflux of bile may cause genotoxic stress. These aspects merit to be tested in wide spectrum of Barrett epithelial tissues.

## Abstract (precis)

The results of the present study provide suggestion that not only acid reflux, but also non-acid reflux of bile may cause genotoxic stress. These aspects merit to be tested in wide spectrum of Barrett epithelial tissues.

## INTRODUCTION

Barrett oesophagus is a premalignant condition and molecular aspects have been characterized from numerous perspectives [1–4]. The major issue with detailing of the pathophysiology of Barrett oesophagus mainly involves the randomness of the exposure of the refluxate, the time of contact of the refluxate with the luminal mucosa and the composition of the refluxate [5–9]. Although it is well known that most refluxates are composed of acid, it is increasingly recognized that non-acid refluxes are also common. This may occur in isolation, or in combination with acid refluxate. Non-acid refluxate can result from ongoing acid suppression therapy in subjects with gastroesophageal reflux disease (GERD). This may also result from mucus reflux. Increasingly, it is also identified that the hypotensive sphincters and other mechanical factors like obesity commonly results in backward flow of contents even from the first part of the duodenum [2]. This results in significant exposure of the luminal oesophageal mucosa to duodenal contents, which include biliary fluid [10].

Mucosal acid sensing mechanisms are known. Recently, bile acid receptors have been described. The bile acid receptors include membrane associated G protein coupled receptors and nuclear receptors [11]. Several bile acid transporters are preset in the intestinal mucosa. It is not known whether the oesophageal stratified epithelium or the metaplastic Barrett epithelium show plastic changes related to expression of the bile acid receptor. Furthermore, because of the irregular and unpredictable exposure of the epithelium to refluxates [12], we utilized a well-based system in which normal oesophageal mucosal flatmounts were seeded on to the wells. A nanospray system was used to expose the top surface to bile acids and consequent changes and epithelial responses were examined.

There are discrepancies and debates regarding the design of an ideal model system to simulate the rheology of refluxate exposure *in vivo* to understand the relevant mechanisms underlying the formation and progression of Barrett epithelium. In the present study, we have utilized periodic exposure of a potential refluxate compound. The major aim of this study was to evaluate whether there is plasticity in expression of the bile acid receptor and whether bile acid exposure leads to genomic stress. Though inflammatory mediators have been identified, chronic inflammatory infiltrates are rarely seen in Barrett epithelium [13–15]. We hypothesized that evanescent stimulation of inflammation may be mediated by gaseous substances like nitric oxide (NO). Inducible nitric oxide synthase (iNOS) is known to mediate inflammation [16,17]. Hence we examined whether iNOS was up-regulated by bile acid exposure. Finally, we examined whether these acute challenge influences genomic changes or induces genotoxic stress.

## MATERIALS AND METHODS

### *In vitro* system for exposure of oesophageal epithelium to bile acids

The study was designed to simulate a system of reflux, not necessarily replicating the time domains during an actual episode of GERD. Whole mounts of lower end of the oesophagus was floated in a multiwall system with the luminal surface facing upwards. Bile acids (mixture of taurocholic and glycocholic acids in equal proportions, p*K*_a1–3_) were sprayed with a picospritzer for 60 s (50 ms pulses at 10 psi) at 0, 6, 8, 12 and 24 h respectively (for example, the sample of 24 h was sprayed earlier as well at the specified time points). Experiments were terminated 1 h after the defined time points. Controls were performed by spraying PBS for the same duration and the number of times (only the chemical composition was different). The flat mounts were floated in physiological balanced salt solution throughout the entire period of the experiments (temperature of the hood maintained at 37°C). Explicit permission and consent was obtained from Institutional Review Board (IRB) to obtain the human tissue samples from the biobank. Oesophagus samples were obtained from local tissue bank and submucosal dissection was performed to separate the mucosal flatmount from the underlying muscularis mucosa using a dissecting microscope (Zeiss), Dumont no. 5 tweezers and iris scissors. All experiments were performed in triplicates and each condition was from an average of three independent experiments. All the subjects had cardiovascular or cerebrovascular deaths (males, age range 45–63 years old) and none had any documented gastrointestinal or oesophageal disease (as observed from the clinical notes).

### Cell-based ELISA

Cell-based enzyme-linked assay was used to identify the plasticity of expression of two key proteins using human epitope specific antibody-based detection systems: bile acid receptor TGR5 (GPBAR1) and iNOS. The whole mounts were fixed with 4% formaldehyde, washed with PBS, quenching and blocking buffer, incubated with primary and secondary antibodies and developed with the provided reagent and read with a plate reader with appropriate stimulation and emission reagent (540/600 and 360/450 nm, protein of interest and normalized proteins respectively) according to the manufacturer's protocols (R&D Biosystems). Readings were obtained in duplicates and averaged to obtain the final results.

### *In vitro* nitric oxide assay

Steady-state assays of *in vitro* NO production was assayed with the cells (of the flatmounts) suspended in the clear microplates in 300 μl total volumes. No formation was identified by an increase in absorbance at 401 nm using the oxyhaemoglobin (HbO_2_) assay. Reaction mixtures were prepared by admixing 50 mM HEPES at pH 7.5, 500 mM NaCl, 1 mM NOS substrate L-arginine, the reducing agent DTT, 10 μM tetrahydrobiopterin, reducing equivalent NADPH (100 μM), additionally supplemented with 1 mM CaCl_2_, 8 μM HbO_2_ and calmodulin (added to enhance the L-arginine oxidative reaction). NO production was monitored as a diminution in the absorbance at 340 nm using the incubation and stimulation conditions mentioned. Control reactions were performed by refraining from adding HbO_2_ to the reaction mixture. Whether NO production was sensitive to iNOS dependent biosynthesis was tested by pre-incubation with aminoguanidine (AG, partial iNOS inhibitor) for an hour prior to the reaction stimulation.

### Nucleo-cytoplasmic separation and ELISA/fluorescence assay for genotoxic stress

Genotoxicity was assessed by examining expression of phosphorylated histone 2AX, γ-H2AX, which is an indicator for DNA double-stranded break [18]. Cellular extracts were spun and nucleo-cytoplasmic separation was made by cold differential centrifugation and the expression assay was performed using the nuclear fractions. Separately, expression of γ-H2AX was also independently assayed by observing immunofluorescence using primary specific antibody (Cell Signaling Technology) and a fluorescent secondary antibody. Fluorescence intensity was assayed in 100 independent nuclei in each group and expressed as a normalized value. The values of the ELISA and fluorescence intensities were correlated for detecting uniformity of expression.

### Cell viability assay

The UCDA was purchased from Sigma–Aldrich. A total of 1×10^4^ cells for each well were seeded in 48-well plates for overnight. The medium was replaced with fresh medium containing the chemical agents and cells were incubated for 12, 24 or 48 h. Cell viability was measured using the MTT assay. Absorbance was measured spectrophotometrically at 570 nm by the Universal Microplate Reader EL800 (BIO-TEK instruments). The experiments were repeated three times.

### Western blotting

To detect protein signals, lysates were electrophoresed on 10–12% PAGE gels. Initial pilot experiments were performed to standardize the time and voltage of separation and each blot were run multiple times to acquire the optimal conditions. After electrophoresis, gels were transferred on to PVDF membranes by running the transfer process overnight. The immunoblots were developed with 1 in 50–1000 dilutions of the antibodies, again with extensive pilots performed to optimize the concentrations, as well as that of the secondary antibodies. Chemiluminescent signal was incubated with the ECL System (GE HealthCare) and signal was developed under dark conditions using Kodak X-ray.

### Statistics

Data were expressed as means S.E.M. Comparisons between multiple groups were performed by ANOVA. Post-hoc analyses were performed by Tukey's HSD test. Paired samples were compared by Student's *t* test.

## RESULTS

### Increased expression of TGR5 in oesophageal epithelium upon acute exposure to bile acid

**Figure 1:**
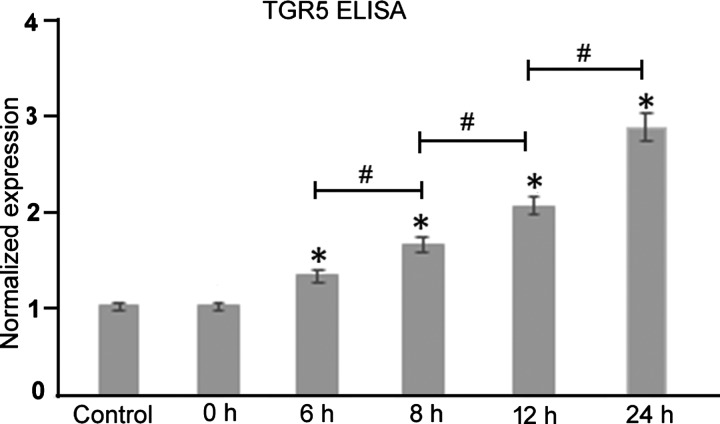
Increased expression of TGR5 in oesophageal epithelium upon acute exposure to bile acid ELISA histograms show increased expression of TGR5 in oesophageal epithelial cells.

**Figure 2:**
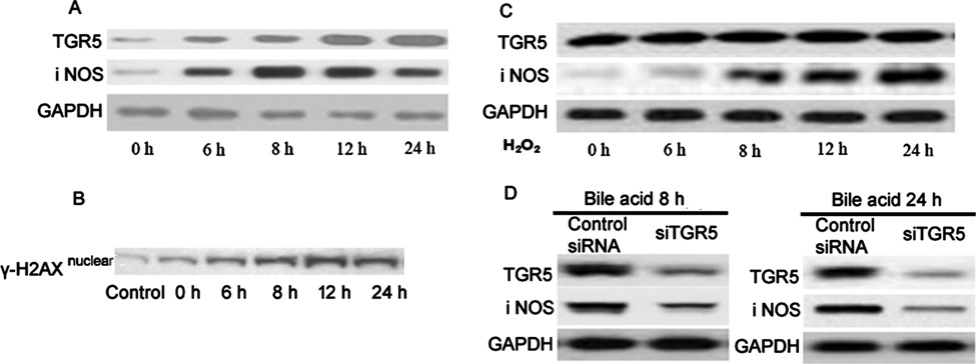
Representative western blots of the temporal changes of expression of different proteins upon controlled exposure to bile acids TGR5 and iNOS are shown in figure **A** (GAPDH loading control). H2AX nuclear expression is shown in figure **B**. The expressions of TGR5 and iNOS by H_2_O_2_ treatments at 0, 6, 8, 12 and 24 h are shown in figure **C** (GAPDH loading control). (**D**) Cells with knockdown of TGR5 show down-regulated expressions of iNOS with bile acid treatments at 8 and 24 h (GAPDH loading control).

Cell-based ELISA assays demonstrated increased expression of TGR5 in oesophageal epithelial cells (1 versus 1 versus 1.33±0.02 versus 1.95±0.01 versus 2.16±0.02 versus 3.18±0.06, Tukey's HSD test; *P<0.01, compared with the control; ^#^*P*<0.01, grouped comparisons) (Figure 1). Because we wanted to detect the membrane-localized receptor, the cell-based assay was adapted. In comparison with controls where PBS was picrosprayed, intermittent exposure of bile acids over a period of 24 h resulted in significant up-regulation of TGR5 (*F*=1066.05, *P*<0.0001, one-way ANOVA). Representative western blots of the temporal changes of expression of TGR5 are shown in Figure 2(A).

### Increased expression of iNOS in oesophageal epithelium upon acute exposure to bile acid

**Figure 3:**
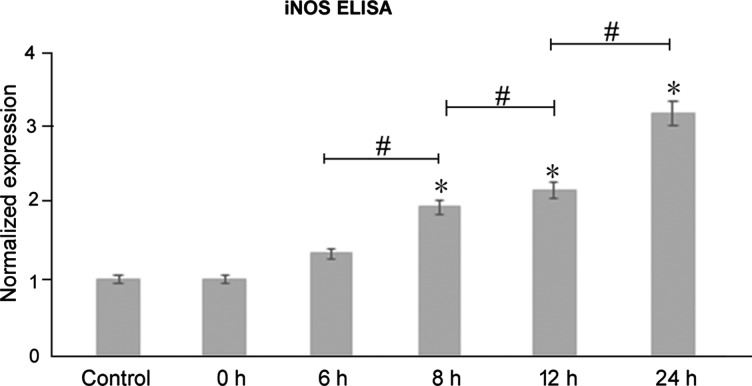
Increased expression of iNOS in oesophageal epithelium upon acute exposure to bile acid ELISA histograms show increased expression of iNOS in oesophageal epithelial cells.

Cell-based ELISA assays demonstrated increased expression of iNOS in oesophageal epithelial cells (1 versus 1 versus 1.34±0.02 versus 1.68±0.01 versus 2.01±0.01 versus 2.96±0.02, mean normalized expression) (Figure 3). In comparison with controls where PBS was picrosprayed, intermittent exposure of bile acids over a period of 24 h resulted in significant up-regulation of iNOS (*F*=285.29, *P*<0.0001, one-way ANOVA; Tukey's HSD test; *P<0.01, compared with the control; ^#^*P*<0.01 grouped comparisons). Representative western blots of the temporal changes of expression of iNOS are shown in Figure 2(A). At high concentrations, some bile acid species induce oxidative stress. To support the hypothesis, we tested whether bile acid affects downstream signalling through indirect effect by oxidative stress. To address this, we treated oesophageal epithelium cells with H_2_O_2_, a well-studied oxidative stress inducer to see whether oxidative stress could regulate the expressions of TGR5 and iNOS. Our results in Figure 2(C) demonstrate that H_2_O_2_ induces the expression of iNOS, consistent with the previous reports. However, H_2_O_2_ cannot induce the TGR5 expression, suggesting although bile acid induces oxidative stress, which activates iNOS, at least bile acid affects the downstream signalling through TGR5, which is independent of oxidative stress. To assess whether the bile acid induced iNOS is partially through the TGR5, we transfected cells with siRNA to specifically knockdown TGR5. Our results in Figure 2(D) show knockdown TGR5 could decrease the expression of iNOS with the treatments of bile acid at 8 and 24 h, indicating TGR5 is required in the bile acid-TGR5-iNOS pathway. Taken together, the above results illustrate bile acid affects downstream signalling at least partially through the non-oxidative stress pathway.

### Increased expression of γ-H2AX in oesophageal epithelium upon acute exposure to bile acid

**Figure 4:**
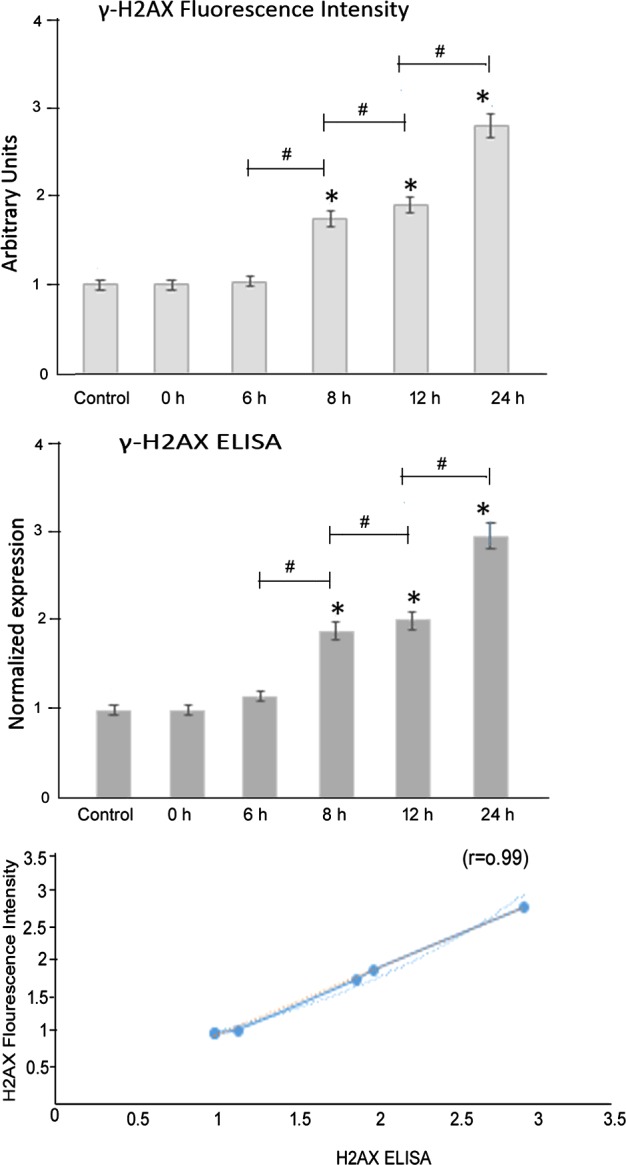
Increased expression of γ-H2AX in oesophageal epithelium upon acute exposure to bile acid Cell-based ELISA assays demonstrated increased expression of γ-H2AX in oesophageal epithelial cells. Fluorescence intensity examination of nuclei demonstrated similar increased expression of γ-H2AX in oesophageal epithelial cells (expressed in arbitrary units). These values were highly correlated (*r*=0.99).

Cell-based ELISA assays demonstrated the increased expression of γ-H2AX in oesophageal epithelial cells. In comparison with controls where PBS was picrosprayed, intermittent exposure of bile acids over a period of 24 h resulted in significant up-regulation of γ-H2AX (*F*=525.38, *P*<0.0001, one-way ANOVA, *P<0.01, compared with the control; ^#^*P*<0.01, grouped comparisons) (Figure 4). Fluorescence intensity examination of nuclei demonstrated similar increased expression of γ-H2AX in oesophageal epithelial cells. In comparison with controls where PBS was picrosprayed, intermittent exposure of bile acids over a period of 24 h resulted in significant up-regulation of γ-H2AX (*P*<0.001, ANOVA). These values were highly correlated (*r*=0.99) (Figure 4). Representative western blots of the temporal changes of expression of nuclear H2AX are shown in Figure 2(B).

### *In vitro* NO production increased in oesophageal epithelial cells upon exposure to bile acids

**Figure 5:**
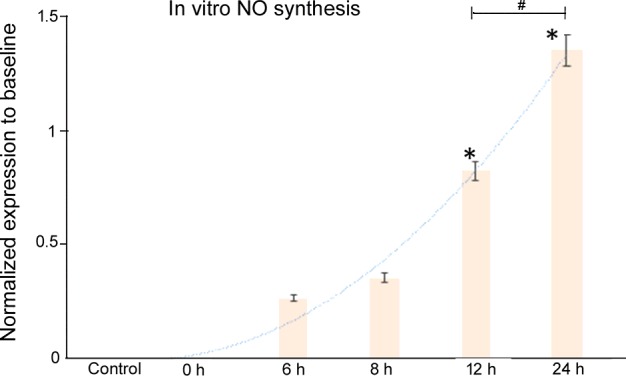
*In vitro* NO production increased in oesophageal epithelial cells upon exposure to bile acids *In vitro* NO production was significantly increased upon exposure to bile acids. At each individual time point, nitric acid production was assayed by the oxyhaemoglobin method. The assays were performed at regular intervals (time points indicated) over a period of 24 h.

*In vitro* NO production was significantly increased upon exposure to bile acids. At each individual time point, nitric acid production was assayed by the oxyhaemoglobin method, with and without incubation with AG, a partial iNOS inhibitor. NO production was significantly elevated upon repeated exposures of bile acids (0 versus 0 versus 0.27±0.02 versus 0.36±0.02 versus 0.83±0.02 versus 1.36±0.08, mean normalized expression as compared with baseline, *F*=61.41, *P*<0.0001, ANOVA) (Figure 5), and production was significantly inhibited by AG (*P<0.01, compared with the control; ^#^*P*<0.01, Student's *t* test, paired sample comparisons).

### UDCA protects oesophageal epithelial cells from the bile acid-induced genotoxic stress

**Figure 6:**
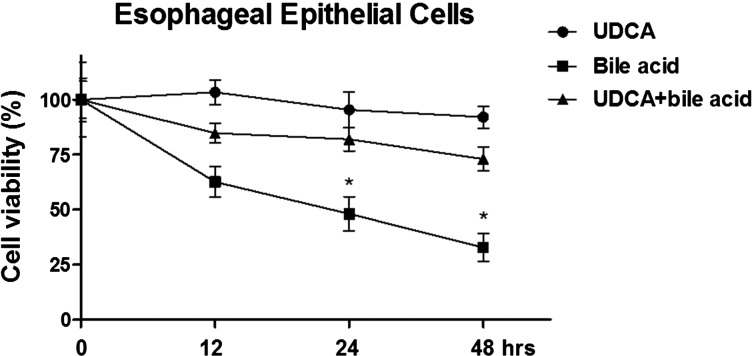
UDCA protects oesophageal epithelial cells from the bile acid-induced genotoxic stress Human oesophageal epithelial cells were treated with bile acid alone, UDCA alone or with the combination of bile acid and UDCA for 12, 24 or 48 h, then the cell viabilities were measured by MTT assay. *P<0.05.

Ursodeoxycholic acid (UDCA) has been utilized in clinical situations like progesterone-induced jaundice. Previous study indicated that UDCA may act as an important pharmacological adjunct agent in acid/bile reflux disease. To test this, we performed cell viability assay to investigate the cytotoxicity from bile acid treatments alone, UDCA treatments alone or with the combination of bile acid and UDCA. Results demonstrated that the bile acid induced cell death by genotoxic stress. However, the combination of bile acid and UDCA could significantly reduce the cells death (Figure 6). (*P<0.05, combination of UDCA and bile acid compared with the bile acid alone; Student's *t* test, paired sample comparisons).

## DISCUSSION

The results of the present study provide the first incipient evidence of direct genotoxic damage caused by exposure of bile acids to the oesophageal epithelium. This was seen as an up-regulation of the phosphorylated histone subtype, which has been well studied as a marker for genotoxic stress and damage due to environmental factors [18–22]. Interestingly, the present study shows that these are mediated by up-regulation of iNOS and synthesis of NO. This may likely explain the relative absence of inflammatory infiltrate of a chronic nature when Barrett's epithelium is examined by routine histopathological methods [13–15]. The increase in NO possesses the potential to diffuse across to the nuclei, causing damage in the nucleosome. In the present study, we did not examine whether DNA damages occur, as has been reported in some earlier studies [23]. Earlier studies have indicated DNA damages in Barrett epithelium by demonstration of increased ploidy and progressive dysplasia [24]. The cytosine or guanine residue damages by bile acids remains the scope of our future studies for direct demonstration of DNA nucleotide damages by exposure of mucosa to bile acids.

One of the interesting observations from our present study is that only transient exposure caused persistence in increased expression of the bile acid receptor, iNOS and enhanced NO production. This indicates that even transient exposure may cause potential damage. We obviously did not observe any change in the epithelial morphology of the floating whole mounts in the short span of the experimental setup. Additionally, we did not examine any of these findings in Barrett epithelium, as it is almost impossible to correlate the nature of the refluxate (its chemical composition) and subsequent metaplastic transformation to metaplastic epithelium.

UDCA has been utilized in clinical situations for the treatment of cholestatic liver diseases through a cytoprotective effect to compensate the toxicity of bile acid. The major mechanisms of action have been illustrated that UDCA protects liver cells against cytotoxicity of hydrophobic bile acids, resulting from modulation of the composition of mixed phospholipid-rich micelles [25]. Moreover, UDCA decreases the concentration of hydrophobic bile acids in the cholangiocytes [25]. Our *in vitro* study demonstrated the combination of UDCA and bile acid could protect oesophageal epithelial cells from the bile acid-induced genotoxic stress, suggesting UDCA might contribute to the clinical development of pharmacological agents against the genotoxic stress in Barrett oesophagus.

We plan to extend our present studies in examining the findings of our *ex vivo* model in actual pathologic biopsy specimens. Furthermore, what remains unresolved is the trigger that probably activates iNOS and examination of the different transcription factors and their network aberration that contributes to ongoing DNA damage as well as dysplastic progression. Given the increasing epidemiology related to obesity and GERD and increased observational reports of oesophageal adenocarcinoma, in addition to the traditionally reported oesophageal squamous cell carcinoma, both in the western countries as well as in the fareast such as China [26], it remains worthwhile to undertake these studies on the pathophysiology of genotoxic stress in Barrett oesophagus.

## Abbreviations

AG: aminoguanidine
GERD: gastroesophageal reflux disease
iNOS: inducible nitric oxide synthase
NO: nitric oxide
UDCA: ursodeoxycholic acid
